# Oral Delivery of Biologics in Inflammatory Bowel Disease Treatment

**DOI:** 10.3389/fbioe.2021.675194

**Published:** 2021-06-03

**Authors:** Wunan Zhang, Cecilia Bohns Michalowski, Ana Beloqui

**Affiliations:** Advanced Drug Delivery and Biomaterials, Louvain Drug Research Institute, Université Catholique de Louvain, Brussels, Belgium

**Keywords:** inflammatory bowel disease, biologics, oral drug delivery, antibody, therapeutic peptide, oligonucleotides, gene therapy

## Abstract

Inflammatory bowel disease (IBD) has been posed as a great worldwide health threat. Having an onset during early adulthood, IBD is a chronic inflammatory disease characterized by remission and relapse. Due to its enigmatic etiology, no cure has been developed at the moment. Conventionally, steroids, 5-aminosalicylic acid, and immunosuppressants have been applied clinically to relieve patients’ syndrome which, unfavorably, causes severe adverse drug reactions including diarrhea, anemia, and glaucoma. Insufficient therapeutic effects also loom, and surgical resection is mandatory in half of the patients within 10 years after diagnosis. Biologics demonstrated unique and differentiative therapeutic mechanism which can alleviate the inflammation more effectively. However, their application in IBD has been hindered considering their stability and toxicity. Scientists have brought up with the concept of nanomedicine to achieve the targeted drug delivery of biologics for IBD. Here, we provide an overview of biologics for IBD treatment and we review existing formulation strategies for different biological categories including antibodies, gene therapy, and peptides. This review highlights the current trends in oral delivery of biologics with an emphasis on the important role of nanomedicine in the development of reliable methods for biologic delivery in IBD treatment.

## Introduction

Inflammatory bowel disease (IBD) is defined as a systemic, autoimmune, relapsing-remitting chronic disease of the gastrointestinal system whose peak incidence commonly comes off in twenties to thirties (Khor et al., 2011; Zhang and Li, 2014; Lichtenstein et al., 2018). Ulcerative colitis (UC) and Crohn disease (CD) are two major forms of IBD (Baumgart and Carding, 2007; Nikolaus and Schreiber, 2007; Strober et al., 2007). Shared with overlapped syndromes of abdominal pain, fever, bowel obstruction, and diarrhea with the passage of blood or mucus, or both, these two forms can be distinguished by diverse pathological features (Nikolaus and Schreiber, 2007). Starting from the rectum, inflammation of UC continuously spreads through the whole colon with superficial mucosal layer affected, while CD is characterized by intermittent inflammation areas alongside the entire gastrointestinal system and inflammatory lesions could invade all layers of the gut wall (Thompson and Lees, 2010).

Posed as a global health threat, the incidence rates of CD and UC varied worldwide between 0.1 and 16 per 100,000 inhabitants and 0.5–24.5 per 100,000 inhabitants, respectively (Kanaan et al., 2012). North America and Europe contracted with the highest popularity of the disease. There are approximately 1.5 million IBD patients in the United States and 2.2 million in Europe (Molodecky et al., 2012). A concerning increasing trend of incidence in Asia has also been reported in recent years (Prideaux et al., 2012). Westernization of lifestyle, diet, genetic susceptibility, microbiome, and abuse of antibiotics have been identified as risk factors for IBD (Ananthakrishnan et al., 2018); however, the precise pathogenesis remains enigmatic, causing an indomitable hindrance of IBD treatment (Shanahan, 2001). Until now, IBD is incurable and abides with compromised therapeutic strategies aiming at alleviation of the syndromes. Current IBD therapy focuses on induction and maintenance of remission in company with endoscopic healing and reduction of hospitalization and surgery (Peyrin–Biroulet et al., 2014).

Therapeutic approaches for IBD treatment should be individualized based on the disease severity, complications, location, and prognosis. According to the guidelines by the American Gastroenterological Association (AGA), European Crohn’s and Colitis Organization (ECCO) together with the European Federation of Crohn’s and Ulcerative Colitis Associations (EFCCA), British Society of Gastroenterology, and National Institute for Health and Care Excellence (NICE), 5-aminosalicylic acid (5-ASA) compounds, comprehending sulfasalazine, mesalamine, and diazo-bonded 5-ASA, is the first-line and standard treatment recommended to treat patients with extensive mild to moderate UC. Depending on the severity of the disease and the localization, oral and rectal administration can be combined, and different dosages and lower or higher doses of the drugs are suggested. Corticosteroids are only recommended in the case of UC patients refractory to 5-ASA, because of their significant adverse effects (Leone et al., 2016; Ko et al., 2019; Lamb et al., 2019; National Institute for Health and Care Excellence, 2019b). Fecal microbiota transplantation could be performed only in the context of clinical trial (Singh et al., 2015). By the moment, there are no recommendations in guidelines for the use of curcumin or probiotics. Regarding the maintenance of the remission, the use of 5-ASA medications seems to be effective, and in long-term use, it might reduce the risk of bowel cancer. The use of azathioprine should be considered when steroids are needed to keep the patient in remission. Those who fail to respond to azathioprine (Leone et al., 2016; Lamb et al., 2019) should be treated with antitumor necrosis factor-α (TNF-α) or tacrolimus; however, the evidence is not so convincing for tacrolimus (Leone et al., 2016). In the case of acute severe UC, the patient should be hospitalized and the first-line treatment is corticosteroids and those who fail to respond should be treated with infliximab or ciclosporin (Lamb et al., 2019).

Regarding CD, the treatment does not follow the same protocol, ECCO, British Society of Gastroenterology, American College of Gastroenterology, and NICE guidelines recommend corticosteroids as first-line treatment, such as budesonide, for mild-to-moderate disease and systemic corticosteroids, such as prednisolone or methylprednisolone, for moderate-to-severe disease treatment (Lichtenstein et al., 2018; Lamb et al., 2019; National Institute for Health and Care Excellence, 2019a; Torres et al., 2020). However, because of the adverse effects and lack of efficacy on the maintenance of remission, corticosteroid treatment is not recommended for long-term remission and after the induction of remission, it can be substituted by thiopurine or methotrexate (Lichtenstein et al., 2018). Monoclonal antibodies are only recommended if patients do not respond to conventional therapy (Lichtenstein et al., 2018; Lamb et al., 2019; National Institute for Health and Care Excellence, 2019a; Torres et al., 2020).

## Current Biologics in Clinical IBD Treatment

The chronicity of the disease and failure of standard treatments trigger a huge amount of scientific inquiry for new treatments allowing maintained remission in patients. Nowadays, biological treatments such as antibodies represent an alternative in IBD treatment and have evidenced reduction in hospitalizations (Shanahan, 2001; Mao et al., 2017) and improvement of life quality, mucosal healing (Peyrin–Biroulet et al., 2014; Cholapranee et al., 2017), and clinical remission. However, biological treatments are still the last option after 5-ASA, corticosteroids, and immunosuppressants. As for antibodies, all of them have the potential of immunogenicity, affecting their efficacy and safety (Danese et al., 2015). Therefore, TNF-α blockers, that have already been widely used in IBD treatment, are administered in combination with immunomodulators such as thiopurines and methotrexate, as it may reduce the formation of antidrug antibody (Molodecky et al., 2012).

In this review, we categorized both large biomolecules such as antibodies and oligonucleotides, and small molecular peptides as biological treatment. For all of those reagents, they target mediators of inflammation, including TNF-α, selective antiadhesion molecules such as α4 integrins, IL-12/IL-23, and interferon (IFN)-γ, Janus kinases (JAK), and sphingosine-1-phosphatase (S1P) receptor inhibitors (Rutgeerts et al., 2009; Hanauer, 2010; Cohen and Dalal, 2015; Danese et al., 2015; Katsanos et al., 2019). In this part, we summarize the biologics in clinical application for IBD treatment (Table 1).

**TABLE 1:** Biologics in the clinics for IBD treatment.

Biologics for IBD in the clinics		
Name	Mechanism	Delivery approach
Infliximab (Bernstein, 2015; National Institute for Health and Care Excellence, 2019b)	Anti TNF-α antibodies	Intravenous
Adalimumab (Bernstein, 2015; National Institute for Health and Care Excellence, 2019b)		Subcutaneous
Certolizumab pegol (Bernstein, 2015; National Institute for Health and Care Excellence, 2019b)		
Golimumab (Probert et al., 2018)		
Natalizumab (National Institute for Health and Care Excellence, 2019a)	Anti α_4_ integrin antibody	Intravenous
Vedolizumab (Bernstein, 2015)	Anti α_4_β_7_ integrin antibody	Intravenous
Ustekinumab (Amiot and Peyrin-Biroulet, 2015)	Anti IL antibody	Intravenous
Tofacitinib (Agrawal et al., 2020)	Non-selective Janus kinase (JAK) inhibitor	Oral

### TNF-α Inhibitors

Four antibodies have been approved as anti-TNF-α treatment: infliximab, adalimumab, certolizumab pegol, and golimumab. Infliximab is administered intravenously at a dose of 5 mg/kg in the weeks 0, 2, and 6 for induction of remission and after every 8 weeks, while adalimumab, certolizumab pegol, and golimumab are administered subcutaneously (SC). Adalimumab is administered at a dose of 160 mg in the week 0, followed by 80 mg after 2 weeks and then 40 mg every 2 weeks. Certolizumab pegol is administered at a dose of 400 mg in the 0, 2, and 4 weeks followed by the same dose every 4 weeks (Bernstein, 2015; National Institute for Health and Care Excellence, 2019b) and finally, golimumab is administered at a dose of 200 mg in week 0 followed by a dose of 100 mg after 2 weeks and after week 6 at a dose of 50 or 100 mg, depending on body weight, every 4 weeks (Probert et al., 2018).

### Antiadhesion Molecules

Antiadhesion therapy is based on the capacity of the molecules to block the integrins on the surface of T cells, preventing cellular homing to the gut (Zundler et al., 2017). Integrins mainly involved in lymphocyte migration are α_2_β_2_ and α_4_ (α_4_β_1_ and α_4_β_7_), and the inflamed gut in IBD patients presents a higher adhesiveness for α_4_ integrins. Two antibodies targeting α_4_ integrins are actually on the market: Natalizumab is an antibody against human α_4_ integrin while vedolizumab is an antibody that is specific for α_4_β_7_ heterodimer (Ghosh and Panaccione, 2010; Danese et al., 2015). Another antibody, etrolizumab is a gut-selective monoclonal antibody against the β_7_ subunit of the heterodimeric integrins α_4_β_7_ and α_*E*_β_7_ and has just completed a phase III study. Most of the guidelines recommend the use of vedolizumab for the maintenance of remission in patients with moderate-to-severe CD given in monotherapy in a dose of 300 mg IV every 8 weeks (Torres et al., 2019).

Alicaforsen is a human antisense oligonucleotide inhibitor of the intercellular adhesion molecule ICAM-1, consequentially blocking leukocyte recruitment (Katsanos et al., 2019). Currently, a phase III study for the treatment of pouchitis (inflammation of the ileal pouch) has been completed in which the drug was administered as an enema in a dose of 240 mg daily for 6 weeks.

### Anti-interleukin Drugs

Ustekinumab is an antibody targeting the p40 subunit, a common component for interleukin (IL)-12 and IL-23. Clinical evidence supported its efficacy in remission maintenance in patients with moderate-to-severe CD who failed to respond to standard or anti TNF-α treatment (Amiot and Peyrin-Biroulet, 2015). The antibody is primarily administered as a single IV infusing over 1 h depending on patients’ body weight and then injected subcutaneously 90 mg SC every 8 weeks. Two other anti-IL-23 antibodies, risankizumab and brazikumab, are in phase II clinical trials (Katsanos et al., 2019).

### Janus Kinase Inhibitors

Tofacitinib, a non-selective Janus kinase (JAK) inhibitor, was approved in Europe in 2018 for UC treatment. Although this small molecular drug can be orally administered, it presents some considerable adverse effects such as thromboembolism, infections, and hyperlipidemia (Agrawal et al., 2020). Filgotinib and upadacitinib are JAK1 inhibitors and have just finished the phase II studies, and phase III randomized clinical trials are currently underway (Katsanos et al., 2019). As tofacitinib, both are orally administered.

### Sphingosine 1 Phosphate Receptor Modulator

Etrasimod and ozanimod are sphingosine 1 phosphate (S1P) receptor modulators. S1P is responsible for the regulation of lymphocyte trafficking, and S1P modulators can interact with the receptor to induce its internalization and inhibit the egress of the lymphocytes from the lymphoid tissue, reducing their accumulation in the inflamed intestinal tissue. Etrasimod has finished the phase II study; it was administered 2 mg orally once daily, and the results showed improvement in Mayo clinical scores (MSC). A phase III study is underway (Sandborn et al., 2020). Ozanimod is also an S1P modulator and was administered orally in a dose of 1 mg/day presenting slightly better results than the placebo group; however, the study was considered not large or long enough to verify efficacy and safety (Sandborn et al., 2016).

## Oral IBD Treatment Strategies

Different pathophysiological signaling pathways have been addressed through new biological treatments. However, limitations of their route of administration bring about slacked clinical compliance, adverse effects, refractory response to treatment, finite efficacy, and high price of treatments, and there is an urgent need to develop new delivery systems. Most of the strategies for conventional IBD treatments are preferred to have a local effect as some of the main pathological manifestations are limited to the intestinal tissue. An oral administration that could directly deliver the drug to the pathogenic site would be desirable (Kesisoglou and Zimmermann, 2005; Dahan et al., 2010), as non-target therapies promote systemic absorption leading to adverse effects and lower efficacy (Rutgeerts et al., 2009; Lee S. H. et al., 2020). On the other hand, oral administration for biologics is challenging since biologics are highly susceptible to the gastrointestinal environment and most of the molecules present limited transport across the intestinal epithelium due to their large size (Anselmo et al., 2019). The main oral strategies for a better performance of biological drugs are chemical modification or formulation-based technologies (Dahan et al., 2010). They are focused on the pathophysiological modifications of the inflamed intestinal tissue such as the production of reactive oxygen species, modification of the pH, mucus production, overexpression of enzymes and receptors, and even changes in the gut microbiota.

**FIGURE 1:**
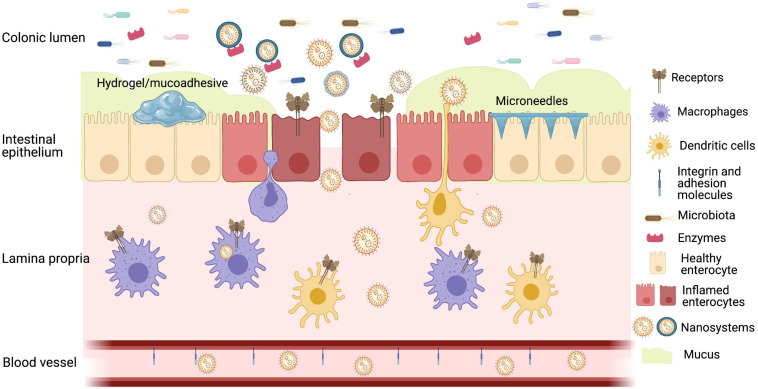
Representative image of oral strategies for colon drug delivery based on inflamed mucosa presenting decreased diversity in microbiota, overexpression of receptors, enzymes, and redox substances, increased permeability, activation of immune system, and decreased mucus barrier. Created with BioRender.com.

Some of the recent strategies (Figure 1) used for oral delivery that could be applied in the treatment of IBD are as follows:

### Polymer-Based Micro/Nanoparticulate Systems

Small nanoparticles (NP) (<200 nm) can reach the intestinal wall with enhanced permeability (Yang and Merlin, 2019). Some pathophysiological characteristics of the inflamed intestinal tissue favor NP accumulation: the increased mucus release seems to improve NP residence on the inflamed site, the destruction of the intestinal barrier increases the NP permeability, and NPs can be taken up by immune cells *via* endocytosis (Beloqui et al., 2016; Li et al., 2020). Other advantages of NPs are that they enable the controlled release profile reducing the adverse effects and dosing frequency. Some of the main important characteristics linked to the development of NPs to target the IBD site are described below.

#### Size

Lamprecht et al. (2001) showed that particles with 100 nm presented better accumulation than 1 or 10 μm particles in the inflamed site in a TNBS model, other studies obtained similar results such as polystyrene particles in a DSS model where particles of 40 and 100 nm had higher accumulation than 0.5 and 1 μm particles (Vong et al., 2012).

#### Surface Characteristics

The mucus in IBD is characterized by the presence of negatively charged colonic mucins. Thus, cationic NPs can enhance surface binding *via* electrostatic interactions and prolong the drug residence time. At the same time, positively charged proteins such as transferrin and eosinophil cationic proteins can also be found in the mucus in inflamed regions which benefits negatively charged particles. As the electrostatic interactions of NPs negatively charged in the mucus are weaker, they can better penetrate the mucus as cationic NPs can be immobilized due to a stronger interaction (Li et al., 2020).

Coating NPs with poly(ethylene glycol) (PEG) can be described as PEGylation and is a common strategy to improve the stability of the NPs in the gastrointestinal tract, likewise improving their absorption. The hydrophilic surface of the NPs created by the presence of the PEG minimizes the mucoadhesion, improving their diffusion (Hua et al., 2015; Suk et al., 2016).

#### Targeting Systems

The presence of a higher concentration of reactive oxygen species on the site of intestinal inflammation as well as inflammatory markers can provide targets for more specific delivery of drugs through surface-modified NPs. Redox-sensitive NPs containing budesonide showed a better accumulation in inflamed tissues and an improved efficacy compared with the drug suspension in a DSS-induced colitis model in male BALB/c mice (Sun et al., 2018). In another study, the accumulation of 40 nm core-shell-type micelles (RPN°) formed by a new redox polymer in the inflamed area were evaluated in a DSS-induced colitis model in ICR mice, showing a greater accumulation in the inflamed area without absorption to the bloodstream (Vong et al., 2012). Different inflammatory markers can also be used as a target such as CD98, CD44, and folate and mannose receptor, all overexpressed on the surface of colon epithelial cells and macrophages and peptide transporter-1 (Pept1) to target macrophages (Yang et al., 2020). Recently, hyaluronic acid has been used to selectively target CD44 (Misra et al., 2015; Lee Y. et al., 2020) while lysine-proline-valine (KPV), a C-terminal peptide fragment of a α-melanocyte-stimulating hormone, can target Pept1 (Wu et al., 2019).

##### pH-dependent systems

Nanoparticles could be coated with a pH-responsive polymer allowing the drug to release in specific pH. Methacrylic acid copolymers (Eudragit^®^) for the colonic delivery of NPs are already well described for non-biological treatments (Beloqui et al., 2014; Naeem et al., 2018; Zhou and Qian, 2018).

##### Enzyme-responsive systems

The microflora present in the colon releases some enzymes to degrade natural polysaccharides, and these polymers can be used accordingly as an alternative coating for NPs to target the colon. Some polymers have already been described in the literature such as guar gum (Kumar et al., 2017), chitosan (Chen et al., 2020), pectin, sodium alginate, and dextran. These polymers are hydrophilic and have the advantage of limited swelling capacity in acidic pH (Rajpurohit et al., 2010).

### Lipid-Based Nanosystems

Liposomes have the advantage of the possibility of encapsulating both lipophilic and hydrophilic drugs, and they can also have their surfaces modified to target the inflamed colon. Solid lipid NPs are more stable systems and better protect the drug providing a more extended release because of the slow degradation of the matrix (Singh et al., 2015; Lee S. H. et al., 2020). Another lipid-based nanosystem is the self-microemulsifying delivery system that is mostly used to encapsulate lipophilic drugs. A surface modification with folate was already performed in this kind of system to improve the solubility of curcumin and also its delivery to the colon (Zhang et al., 2012).

### Natural Nanomedicine

Recently, NPs synthesized with natural products have been developed to overcome problematic issues of polymeric NPs such as toxicity and scale up for their synthesis (Yang et al., 2020). Some of the examples are ginger-derived nanolipids developed for short-interfering RNA (siRNA) delivery against CD98 (Zhang et al., 2017) and *Lycium barbarum*-lipid-based NPs which present excellent anti-inflammatory properties (Zu et al., 2020). Other natural nanosized structures of interest are the exosome like, which can present biological effects likewise transport property. As an example, grape exosome-like NPs could induce protection against colitis in a DSS-induced mice model *via* the proliferation of intestinal stem cells (Ju et al., 2013).

### Hydrogel-Based Systems

Nanogels are a platform that could protect the drug or nanosystem from the acidic pH of the upper part of the GIT releasing the content once it arrives in the intestinal tract. Laroui et al. (2010) developed an alginate-chitosan hydrogel for the delivery of a tripeptide (KPV) and also CD98 siRNA/PEI-loaded NPs (Laroui et al., 2014), while Knipe et al. (2016) developed an enzyme and pH-responsive microencapsulated nanogel for the delivery of siRNA-targeting TNF-α.

### Mucoadhesive Patches

They consist of an enteric capsule containing layers of a mucoadhesive film containing the drug in the middle layer and an external backing layer that protects the drug from degradation by the luminal proteolytic enzymes (Eiamtrakarn et al., 2002). This patches allows the drug to diffuse in a unidirectional way, by creating a higher concentration matrix (Anselmo et al., 2019).

### Genetic Engineering Approaches

Genetic engineering approaches include the use of genetically modified bacteria (probiotics) that produce and secrete biotherapeutics in the disease site under the stimuli of a biomarker such as nitric oxide in CD. These bacteria can also accumulate at the sites containing higher concentrations of the biomarker (McKay et al., 2018).

### Traditional Pharmaceutical Forms

The use of reversible permeation enhancers is one of the strategies used nowadays to improve the absorption of macromolecules, and one of the substances that are widely used is chitosan. Another strategy is the use of natural mucoadhesive polymers such as gelatin, pectin, chitosan, and others (Mantaj and Vllasaliu, 2020). Modified release systems deliver the drug to the site of action in a controlled manner, and some formulations are already in the market as conventional treatments (Doherty and Peppercorn, 2009; Kornbluth, 2015). One of the new technology developed is the “microneedle pills” that were developed to only perform a physical change with no need to modify the drug or the requirement of a specific formulation (Schoellhammer et al., 2016). The microneedles attach to the intestinal epithelium and break it, facilitating the physical absorption of the drug (Anselmo et al., 2019). Traverso et al. (2015) did an *in vivo* proof of concept in swine using as a model macromolecule insulin, and the systemic delivery of the drug was analyzed after serial injections in different sites: gastric, duodenal, and colonic mucosa. The study proved that the system could improve the bioavailability of the macromolecule in all of the cases and that the system could be administered and excreted from the GI tract safely (Traverso et al., 2015).

### Intestinal Microbiota

Dysbiosis, or the alteration of gut microbiota, is one of the factors present in IBD. Healthy individuals present a significant different microbiota compared with individuals with IBD, who present a decrease in commensal bacteria diversity, abnormal composition, and altered spatial distribution (Khan et al., 2019; Guo et al., 2020). Studies showed that some metal NPs that could regulate the intestinal microbiota reduced IBD symptoms (Yang et al., 2020). Nowadays, synbiotics, a combination of pre- and probiotics are being tested for IBD treatment; however, only few studies are available (Khan et al., 2019).

## Oral Delivery of Biologics in IBD Treatment

### Monoclonal Antibodies

The dysfunction of different cytokines in IBD does not only drive intestinal inflammation but also is associated with complications of IBD such as intestinal stenosis, fistula formation, and colitis-associated neoplasias (Neurath, 2014). Respectively, CD is usually outlined with an elevated level of IL-12, IL-23, interferon (IFN)-γ, and IL-17 secreted by T helper-type (Th)1 and Th17, whereas UC is usually designated with escalated Th2 production including IL-13, IL-5, and IL-9 (De Souza and Fiocchi, 2016). Antibodies blocking proinflammatory cytokines such as TNF, IL-12/IL-23p40, IFN-γ, IL-6R, IL-11, IL-13, and IL-17A have been studied as potential modalities for IBD (Neurath, 2014; De Souza and Fiocchi, 2016; Guan and Zhang, 2017). This concept was not validated until clinical approval of infliximab in 1997 (Markham and Lamb, 2000).

TNF-α is initiatively secreted as a 26-kD transmembrane protein (tmTNF), subsequently cleaved off by TNF-α-converting enzyme (TACE) to generate soluble TNF. Soluble TNF-α further reacts with different TNF-α receptors (TFR) to initiate a series of inflammatory reactions (Levin et al., 2016). Anti-TNF-α antibodies block the interaction between TNF-α molecules and TNFR1 and TNFR2 as well as soluble TNF-α receptors (sTNFR), neutralize TNF-α–mediated proinflammatory cell signaling, and inhibit the expression of inflammatory genes (Nielsen and Ainsworth, 2013). However, etanercept, a TNF receptor fusion protein to nullify sTNF-α and inapt to bind tmTNF-α is not effective in IBD patients (Sandborn et al., 2001), implying more convoluted mechanisms are involved in anti-TNF-α antibody therapy for IBD treatment. Recent publications substantiated that infliximab and other anti-TNF-α antibodies reduced T cell proliferation capability through the binding between the drug and tmTNF-α on activated T cells (Vos et al., 2012). Once bound, a distinct macrophage subset (CD14 + HLADR + CD206 +) was induced by the interaction between Fc region and antigen-presenting cells with specific immunosuppressive capacities. CD206 + then inhibited T cell proliferation (Vos et al., 2011). These CD206 + macrophages also had wound-healing properties and were able to cause mucosal healing in an *in vitro* model (Vos et al., 2012).

The complex mechanisms behind antibody therapy accelerated the establishment of new types of antibodies for IBD. After the approval of infliximab, six sequential monoclonal antibodies were approved as previously described. These monoclonal antibodies rendered crucial alternatives, especially to patients who failed to respond to the conventional drug. However, 10–40% of the patients do not respond to anti-TNF-α antibody treatment during precursory induction therapy, and 24–46% of patients have a secondary loss of response in the first year of treatment (Ben-Horin and Chowers, 2011). This partially contributes to the antibody’s immunogenicity, which can lower plasma drug concentration by inducing antidrug antibody (Kennedy et al., 2019). A colon-targeting delivery *via* the oral route is competent to exempt patients from long-term injection pain. With lowered systemic drug concentration, it is capable of downsizing the output of antidrug antibodies and reduce or even counterpart ADRs of biologics, implicitly providing a safer platform with improved therapeutic efficacy. However, until now, biologics can only be administered either subcutaneously or intravenously. At the moment, multiple oral approaches are under development for monoclonal antibody delivery for IBD treatment.

Most antibodies are vulnerable, confronting stomach acid and trypsin, chymotrypsin, or other enzymes abundant in the intestine and degrade before arriving at the inflammatory colon area (Yun et al., 2013). Antibody modifications can enhance their survival in the gastrointestinal tract. AVX-470 was generated by purifying immunoglobulin (Ig) from the colostrum of cows immunized with recombinant human TNF (Bhol et al., 2013). This chimeric antibody can efficiently increase proteolysis resistance and ride out the gastrointestinal tract owing to the virtue of immunoglobulins from bovine colostrum (Roos et al., 1995). In one early animal study involving acute dextran sulfate sodium (DSS)-induced colitis mice model, chronic DSS-induced mice model, and 2,4,6-trinitrobenzene sulfonic acid (TNBS)-induced colitis mice model, mice orally delivered with AVX-470 (mAVX-470) reduced colitis severity observed by endoscopy. Particularly, in chronic DSS-induced colitis models, mice treated with mAVX-470 demonstrated recovery of both body weight loss and colon length after DSS treatment, implying amelioration of colonic inflammation (Bhol et al., 2013). Later in 2013, AVX-470 was under first phase clinical trial to assess its safety, pharmacokinetics, immunogenicity, and preliminary efficacy in patients with active UC. Across all AVX-470 doses, 25.9% of patients achieved clinical response compared with 11.1% on placebo, with the greatest improvements in the 3.5-g/day group associated with proximal colon endoscopic improvement. No severe side effect was observed in all 37 patients within 28 days. Furthermore, oral-delivered AVX-470 also demonstrated reduced systemic exposure in the blood (Harris et al., 2016). However, this study did not attempt to distinguish between AVX-470 and endorsed infliximab. To the best of our knowledge, no further clinical study has been conducted. V565 is a 12.6-kDa anti-TNF-α heavy chain variable domain antibody. It is isolated from a phage library produced from lymphocytes of a human TNF-α hyperimmunized llama and engineered to be resistant to intestinal and inflammatory proteases while retaining the TNF-α neutralizing potency against both soluble and membrane forms of human TNF-α (Crowe et al., 2017; Nurbhai et al., 2019). Opposite to AVX-470, V565 is unable to survive in acidic stomach environment and requires sustenance from oral delivery vehicles. In one *in vivo* study, DSS-induced colitis mice were administered with 140 μg V565 by oral gavage after given a gastroprotective vehicle (0.1 M NaHCO_3_ containing 400 mg/ml Marvel milk). V565 transited well through the mouse GIT reaching high concentration within the lower GI tract and feces up to 7 h postdosing. In this study, treatment with V565 also inhibited inflammatory cytokines’ production with maximal inhibitory effects similar to those achieved with the clinical positive control antibody adalimumab *in vitro* (Crowe et al., 2018). In another study based on the cynomolgus monkey model, oral V565 tablet coated with Eudragit enteric coat survived in the stomach and dissolved in the small intestine. Despite very high V565 concentrations in these monkeys’ intestines, serum V565 was only found at deficient levels because V565 poorly traversed the intact epithelium of normal animals (Crowe et al., 2019). The potential of V565 as oral treatment has been tested in both CD and UC clinically. In a phase I clinical study, local and systemic pharmacokinetics were investigated in four patients with ulcerative colitis. Eudragit-coated V565 was encapsulated in hydroxypropyl methylcellulose (HPMC) capsules. Overall, this study showed that oral-delivered active V565 reaches IBD disease sites in high concentrations and can bind to V565 TNF-α^+^ cells in UC lesions (Nurbhai et al., 2019). In another phase I clinical study, 47 patients with CD were involved in investigating tolerability and safety of oral V565, and V565 is currently under phase II clinical study in Europe and North America (Crowe et al., 2017).

Limited cases of antibody-loaded NPs were reported for IBD treatment in recent decades. Nanoparticles with a size of 100 nm illustrated a higher accumulation in the colon (Lamprecht et al., 2001). In one study, tannic acid and poly(ethylene glycol)-containing polymer self-assembled supramolecular NPs with a particle size of approximately 100 nm were utilized for anti-TNF-α antibody delivery (Wang et al., 2020). DSS effect on colon length can be suppressed by the treatment of oral-delivered infliximab nanoparticles. The biodistribution study showed a minimized systemic exposure with low accumulation in the liver (Wang et al., 2020). Liposomes and other bilayer structure vesicles (Lee and Feijen, 2012; Akbarzadeh et al., 2013; Zhang et al., 2019) are broadly applied for drug delivery purposes. Jung Min Kim and others reported a series of infliximab-loaded liposomes for oral IBD treatment. As a result, infliximab-loaded liposomes had better colitis improvement than the control group alongside with remarkably decreased TNF-α level in a DSS-induced mouse colitis model (Kim et al., 2020).

In recent studies, scientists endeavored to use engineered bacteria as vectors for therapeutic agents (Hosseinidoust et al., 2016). *Lactococcus lactis* (*L. lactis*) was originally isolated from plants where it maintained dormant and only became active and multiplied in the GIT after being consumed by ruminants (Song et al., 2017). *L. lactis* is regarded as a safe vector. It cannot, on ingestion, invade the tissues, nor does it ever cause infection in humans and, at the same time, convey any health benefit (Steidler and Rottiers, 2006). After the report of using Lactobacilli as live vaccine delivery vectors in 2002, *L. lactis* has been recognized as a compelling candidate to introduce foreign antigens or antibodies (Seegers, 2002). Genetically engineered *L. actis* secreting IL-10 was first reported for IBD treatment in mice and pig model (Steidler et al., 2000, 2003). Its oral formulation, AG011, have been getting involved in multiple clinical trials. Phases I and II clinical studies have been conducted in patients with moderate UC. In another phase I clinical trial, AG011 was reported to reduce systemic adverse drug reactions in patients with CD (Braat et al., 2006). Genetically engineered *L. lactis* provided a robust oral delivery platform in IBD therapy. Up until now, versatile cytokines or antibodies have been recombined into *L. lactis* for oral IBD treatment. Vandenbroucke et al. (2010) engineered *L. lactis* to secrete monovalent and bivalent murine (m)TNF-neutralizing nanobodies as therapeutic proteins. In their study, oral administration of nanobody-secreting *L. lactis* made successful local delivery of anti-mTNF nanobodies at the colon and significantly reduced inflammation in mice with (DSS)-induced chronic colitis. In addition, this approach was also successful in improving established enterocolitis in IL-10^–/–^ mice (Vandenbroucke et al., 2010). In another study, *L. lactis* carrying the scFv expression vector was administered by gavage to mice with DSS-induced colitis. After 4 days of treatment, animals showed a significant improvement in histological score and disease activity index compared with those of untreated animals (Chiabai et al., 2019). Namai et al. (2020) showed *L. lactis* was able to deliver IL-1 antagonists. It was exemplified that orally delivered *L. lactis* secreting IL-1 antagonist was able to alleviate inflammation in an acute DSS-induced colitis mouse model (Namai et al., 2020).

### Therapeutic Peptides

Therapeutic peptides represent a unique class of pharmaceutical compounds containing intrinsic signaling molecules for many physiological functions. It presents a new opportunity for therapeutic intervention that closely mimics natural pathways (Lau and Dunn, 2018). Understanding of the efficacy of peptides is only comprehended in the recent two decades. Even though various peptides have been used for IBD treatment (Table 2), the oral delivery systems for these drugs remain deficient. In this section, we will present existing oral delivery systems for therapeutic peptides in IBD.

**TABLE 2:** Therapeutic peptides applied in IBD treatment.

Therapeutic peptides applied for IBD treatment		
Name	Mechanism for IBD	Delivery approach
KPV (Dalmasso et al., 2008)	Interaction with PepT1	Oral
VIP (Lakhan and Kirchgessner, 2010; Dulari et al., 2020)	Modulates the immune system by binding to two G-protein-coupled VIP receptor	Oral
Cortistatin (Gonzalez-Rey et al., 2006)	Binding to receptor for the growth hormone secretagogue ghrelin	Intrarectal
Ac2-26 (Li et al., 2019)	Inhibitor of NF-κB	Oral delivery
GLP-2 (Ivory et al., 2008)	Inhibiting ERK1/2, JNK1/2, NF-κB signaling pathways, and SOCS in STAT-3 signaling	Subcutaneous
WKYMVm (Kim et al., 2013)	Inhibit the production of proinflammatory cytokines such as TNF-α and interleukin (IL)-1β	Subcutaneous
Cathelicidin (Koon et al., 2011)	Toll-like receptor	Intracolonic
Adrenomedullin (Ashizuka et al., 2013)	Downregulation of inflammatory cytokines	NA
Tyr-Pro-D-Ala-NH2–DI-1 (Salaga et al., 2018)	Regulate GLP-2 level	Intracolonic

KPV, a tripeptide composed of Lys-Pro-Val, possesses anti-inflammatory properties associated with PepT1 and was lately regarded as a candidate for IBD therapy (Dalmasso et al., 2008; Viennois et al., 2016). KPV was reported to ease colitis by adding in drinking water at a concentration of 205 μg/day in the DSS-induced mice model (Dalmasso et al., 2008). To fully exploit its therapeutic efficacy, several colitis-targeting systems were developed to colocalize KPV with inflammation tissue. Polymeric NPs loaded with KPV were reported by Laroui et al. (2010). In this study, KPV was encapsulated in NPs composed of polylactide (PLA) and PVA using a double-emulsion/solvent evaporation method. KPV-loaded NPs can be taken up *via* endocytosis, causing 12,000-fold lower delivered KPV concentration than that of KPV in free solution, with therapeutic efficacy (Laroui et al., 2010). Drug delivery systems composed of multiple components were also reported. Hyaluronic acid (HA) can selectively recognize CD44 receptors overexpressed on the surface of colonic epithelial cells and macrophages in colitis tissues. Xiao et al. (2017) fabricated HA-functionalized polymeric NPs for KPV which were further loaded into a chitosan-based hydrogel. This HA-KPV-NP/hydrogel system has the capacity to release HA-KPV-NPs in the colonic lumen and subsequently penetrate colitis tissues and enable KPV to be internalized into target cells (Xiao et al., 2017). In some studies, KPV was utilized as a part of the drug carrier. Wu et al. (2019) developed a colon-specific delivery system—PLGA-KPV/MMT/CS multifunctional medicinal nanoparticles loaded with cyclosporine A (CyA). In this study, KPV is utilized as a ligand for PepT1 to achieve targeting delivery. After being treated with the CyA-PLGA-KPV/MMT/CS nanoparticles (PKMCN), the mice treated with DSS-induced colitis exhibited significant improvements in body weight, colon length, and disease activity index (Wu et al., 2019).

Vasoactive intestinal peptide (VIP) is a 28-amino acid neuropeptide isolated from the intestine (Pozo et al., 2000). Elevated total VIP colonic level was found by radioimmunoassay in the CD tissues due to its modulation effect on cytokines (Abad et al., 2012; Zhang et al., 2016). Studies have validated the efficacy of VIP for IBD therapy in TNBS-induced colitis mice model *via* i.p. injection (Abad et al., 2003). Research on its oral delivery remains insufficient and only *in vitro* results are approachable. Dulari et al. (2020) constructed sterically stabilized micelles (VIP-SSM) for VIP’s oral delivery and conducted *in vitro* release study of this potential oral formulation. However, further *in vivo* studies are required to validate its potency.

Ac2-26 is an annexin A1 N-terminal-derived peptide which can inhibit various aspects of the inflammatory response. Li et al. (2019) designed a reactive oxygen species responsive (ROS) delivery system for Ac2-26 which can selectively respond to abundant ROS in inflammation area and accomplish colitis targeting effect. In this study, orally delivered Ac2-26 effectively decreased the expression of proinflammatory mediators and reduced inflammation symptoms in both acute and chronic DSS-induced mice model (Li et al., 2019).

### Oligonucleotides

Therapeutic oligonucleotides are synthesized nucleic acids interfering with the pathogenesis. Multiple forms of oligonucleotides are being applied in medical science, entailing different molecular mechanisms, including both inhibition of the translational process of messenger ribonucleic acid (mRNA) transcripts and mimicking bacterial deoxyribonucleic acid (DNA) which can activate cellular targets for immunomodulation (Scarozza et al., 2019; Smith and Zain, 2019). mRNA-silencing oligonucleotides are the main platforms studied for IBD treatment. This review will focus on introducing mRNA-silencing oligonucleotides in IBD treatment: siRNA and antisense oligonucleotide (AON).

Short-interfering RNAs are double-stranded RNA (dsRNA) which can suppress gene expression through a highly regulated enzyme-mediated process called RNA interference (RNAi), an endogenous pathway for posttranscriptional silencing of gene expression (Reynolds et al., 2004; Kanasty et al., 2013). dsRNAs are chopped up into 19–23 bp duplexes RNAs. One strand of these duplex RNA then binds to the RNA-induced silencing complex (RISC) and is unwind in an ATP-dependent manner. RISC mediates sequence-specific binding of these dsRNAs to a corresponding mRNA and catalyzes the cleavage and destruction of the mRNA by the enzyme slicer, enabling gene-specific silencing (Voinnet, 2005; Devi, 2006). siRNA in IBD treatment is designed to block mRNAs transcribing proinflammatory cytokines. However, siRNA can only activate after successful transfection which sets two hurdles on its further application (Wang et al., 2010). Firstly, siRNA is unstable in the circulation system with a very short plasma half-life of about 10 min (Takahashi et al., 2009). More importantly, siRNA can be only uptaken by targeting cells *via* endocytosis, and during its internalization, lysosomes can degrade siRNA (Tseng et al., 2009). Thus, colitis-targeting oral delivery is required to balance their ability to protect siRNA from degradation in cytoplasma and plasma and prolong its pharmacokinetics, to colocalize siRNA in inflammatory lesions which prevent potential interference of siRNA on healthy normal tissue and to guide siRNA to survive lysosome. These presurmises determined the extremely challenging nature of the oral siRNA delivery system for IBD treatment. Targeted natural NPs from edible plants are utilized for siRNA delivery in oral IBD treatment (Yang and Merlin, 2020). Active components in ginger such as 6-gingerol and 6-shogaol illustrated anti-inflammatory effects (Grzanna et al., 2005). Zhang et al. (2016) reported that ginger-derived nanoparticles (GDNPs) can ameliorate colitis syndromes and enhance the wound-healing process. The same group prepared ginger-derived lipid vehicles (GDLVs) out of GDNPs for siRNA delivery. GDLVs loaded with siRNA-CD98 effectively reduced the expression of CD98 in the colon and demonstrated a great potential for IBD treatment (Zhang et al., 2017). Kriegel and Amiji (2011a) reported a nanoparticle-in-microsphere oral system. TNF-α specific siRNA was encapsulated in type B gelatin nanoparticles and further entrapped in poly(epsilon-caprolactone) (PCL) microspheres. Successful gene silencing led to decreased colonic levels of TNF-α and suppressed expression of other proinflammatory cytokines in DSS-induced colitis mice model (Kriegel and Amiji, 2011a). The same delivery system encapsulated with a combination of siRNA duplexes specifically targeted against TNF-α and cyclin D was also reported (Kriegel and Amiji, 2011b). Mitogen-activated protein kinase kinase kinase kinase 4 (Map4k4) has been demonstrated to be a key upstream mediator of TNF-α action (Aouadi et al., 2009). Zhang et al. (2013) reported that galactosylated trimethyl chitosan–cysteine (GTC) NPs for oral delivery of a Map4k4 siRNA (siMap4k4) activated macrophages and demonstrated therapeutic efficacy in DSS-induced colitis mice model. Knipe et al. (2016) designed a platform consisting of microgels composed of poly(methacrylic acid-co-*N*-vinyl-2-pyrrolidone) (P[MAA-co-NVP]) cross-linked with a trypsin-degradable peptide linker. This pH-responsive microencapsulated nanogel can protect TNF alpha-siRNA against gastrointestinal environment (Knipe et al., 2016). Wilson et al. (2010) developed thioketal nanoparticles for oral delivery of anti-TNF-α siRNA. Nanoparticles can protect siRNA from degradation in the stomach until it reacts with abnormally high ROS in inflammation areas in the colon more precisely colocalization of therapeutic gene and inflammatory lesion.

Antisense oligonucleotide are single-stranded DNA molecules binding to complementary mRNA by base pairing and induce cleavage of targeted mRNA by ribonuclease H, an enzyme that degrades RNA in RNA-DNA duplexes, leading to decreased gene expression (Kole et al., 2012; Di Fusco et al., 2019). In contrast to siRNA, which tolerates only minimum modifications to remain its efficacy, more extensive chemical modifications do not abrogate RNase H activity on antisense DNA-RNA complexes (Kole et al., 2012). Phosphorothioate (PS) is one of the most common methods to stabilize antisense oligonucleotide which provides increased resistance to the nucleases thereby extending AON’s half-lives (Crooke, 2007). More importantly, PS induces protein adsorption on the AON strand which further prevents rapid AON clearance by glomerular filtration (Crooke, 2007). Mongersen (GED0301) is a formulation containing a 21-base single-strand phosphorothioate oligonucleotide targeting SMAD7, an intracellular protein associated with abnormal cytokine deficiency in CD patients (Monteleone et al., 2001, 2015; Ardizzone et al., 2016). A preclinical study demonstrated that mongersen facilitates TGF-β1–mediated suppression of colitis after oral administration with bicarbonate in the TNBS induced-colitis mice model (Boirivant et al., 2006). In several clinical studies, morgersen is encapsulated in the modified tablet to achieve controlled release in the lumen of the terminal ileum and right colon (Ardizzone et al., 2016). In a double-blind, placebo-controlled, phase 2 trial, 188 patients with moderate-to-severe CD were enrolled in this study. The rate of clinical response was significantly greater among patients receiving 10 mg (37%), 40 mg (58%), or 160 mg (72%) of mongersen than among those receiving placebo (17%) (Monteleone et al., 2015). However, in the report of the newest phase 3 clinical trials, proportions of patients achieving clinical remission at week 52 were similar among individual morgersen groups and placebo at study termination (Sands et al., 2020). AONs with other pharmacological targets are also investigated though very few studies have focused on their oral delivery (Table 3). To the best of our knowledge, only one NP has been designed for oral delivery of AON in IBD treatment. In this study, AON against TNF-α was delivered *via* polysaccharide-based NPs. The oral-delivered NPs reduce TNF-α production by 36.4% in DSS-induced mice model (Duan et al., 2019).

**TABLE 3:** Antisense oligonucleotides in IBD treatment.

Antisense oligonucleotides for IBD treatment	
Target of AONs	Administration approach
Intracellular adhesion molecule-1 (alicaforsen) (Jairath et al., 2017)	Intravenous, rectal
Inhibition of Smad7 (mongersen) (Boirivant et al., 2006)	Oral
NF-κBp65 (Murano et al., 2000)	Intracolonic
Blocking CD154/CD40 interactions (Gao et al., 2005)	Intracolonic
TNF-α (Duan et al., 2019)	Oral

### Gene Therapy

Gene therapy is described as products that mediate their effects by transcription and/or translation of transferred genetic material and/or by integrating into the host genome (Wirth et al., 2013). It was hypothesized that in contrast to protein-based drugs that may require repeated infusion, gene-based therapies might maintain sustained production of endogenous proteins, such as clotting factors in hemophilia (High and Anguela, 2016; Dunbar et al., 2018). Like any other oligonucleotides, gene therapy components will degrade in physiological condition and cannot transfect targeted cells spontaneously. Vectors are required for successful gene therapy delivery. Viruses are naturally designed to repose their nucleic acid into living cells. It was reported that a topically delivered pseudo-typed lentivirus vector encoding murine IL-10 successfully penetrated local mucosal tissue and had therapeutic benefits in DSS-induced mice model (Matsumoto et al., 2014). In another study, recombinant adenoviruses are used as the vector for peroxisome proliferator-activated receptor (PPAR)γ gene therapy (Katayama et al., 2003). Activation of PPARγ in the intestinal tissues plays an anti-inflammatory role against colitis (Su et al., 1999). In this study, low tissue levels of PPARγ can be reversed by gene therapy allowing for a therapeutic response *via* i.p. injection (Katayama et al., 2003). No evidence supported viral vectors can be delivered *via* oral administration according to the best of our knowledge. This may be partially due to their immunogenicity and susceptibility toward the gastrointestinal environment including the gastrointestinal lumen and the intestinal mucosa. Scientists also used bacteria such as *E. coli* as alternatives for oral-delivered gene therapy (Castagliuolo et al., 2005). Palffy et al. (2011) selected bacteria, *Salmonella*, to carry plasmids with genes encoding Cu–Zn superoxide dismutase and an N-terminal deletion mutant of monocyte chemoattractant protein-1 for oral delivery. This approach alleviated colitis in DSS-induced mice models (Palffy et al., 2011). Non-viral vectors such as lipid nanoparticles (LNP) are another major tool for gene therapy (del Pozo-Rodríguez et al., 2016). By selecting biocompatible materials, those non-viral vectors can elude the immunogenicity of viral vectors. Bhavsar and Amiji (2008) reported an oral-delivered gene therapy using non-viral vectors. This study used a nanoparticle-in-microsphere oral system (NiMOS) loaded with murine IL-10-expressing plasmid DNA in type-B gelatin nanoparticles. As a result, local-transfected IL-10 was very effective in reducing the levels of proinflammatory cytokines and demonstrated therapeutic effect in the TNBS-induced colitis model (Bhavsar and Amiji, 2008).

## Future Perspectives

Colitis targeting *via* the oral route has demonstrated favorable properties including increased patients’ compliance, reduced ADRs, and enhanced drug concentration in inflammatory lesions. Current oral delivery systems for biologics in IBD therapy are designed to meet two fundamental requirements: secured transition of biologics in the gastrointestinal environment and decreased systemic exposure while maintaining therapeutic efficacy. Yet only four oral biologic formulations (AVX-570, V565, AG011, and mongersen) have been transited into clinical study, and none of them have been approved. In this section, we provide with several perspectives that may guide the advancement of oral delivery system in the future study.

With a more comprehensive understanding on the pathogenesis process of IBD, more types of biologics are added into the therapeutic armamentarium. However, current oral delivery systems are scarce to cover all these reagents. For instance, most studies on oral-delivered antibodies were focused on anti-TNF-α antibodies, and to the best of our knowledge, no oral delivery system has been designed for antiadhesion or anti-IL antibodies. The similar unmet requirement is observed for therapeutic peptides. Furthermore, the current design strategy for oral-delivered biologics carry two major drawbacks: (1) the complexity of an oral delivery system can create barriers to their transition to clinical study and industrialized fabrication and (2) oral drug delivery systems for antibodies should keep a large drug capacity to neglect the effect of antidrug antibody, which, oppositely, is undermined by the encapsulation procedure. Some of the aspects can be enhanced. NPs for biologics in IBD treatment passively accumulate in the colon *via* a size-dependent mechanism. As mentioned in the former section, 100 nm NPs demonstrated the highest accumulation in the colon whereas many of NPs designed for oral delivery have a size two- to fivefold larger. Besides, NPs can be modified with different molecules to achieve active targeting such as lectin to selectively bind to the Thomsen–Friedenreich (TF) blood group antigen in the colon (Ryder et al., 1998; Moulari et al., 2014). *L. lactis* is a substantial tool for the oral antibody delivery in IBD therapy. Accordingly, it can also carry other biologics such as therapeutic peptides (Agarwal et al., 2014). In addition, new formulations for oral biologics should be added. Some topical formulations demonstrated great potential in IBD treatment and can be transferred into oral delivery. Adhesive patches are able to adhere to mucus and was used widely as a topical delivery system for the oral cavity (Nafee et al., 2003). Oral-delivered intestinal adhesive patches were developed for insulin delivery and can be an interesting candidate for IBD therapy (Gupta et al., 2013). Rectal foam has been clinically applied in IBD treatment for the delivery of hydrocortisone and mesalazine. Its expansion and retention ability provide sufficient covered area and enhanced drug concentration in the interface between foam and inflammatory tissue (Arévalo-Pérez et al., 2020). Oral-delivered foam is an interesting concept with very rare evidence to validate (Haznar-Garbacz et al., 2019). So far, oral delivery of biologics is still an unmet medical need for IBD treatment requiring further development.

Inflammatory bowel disease involves very convoluted pathogenesis, and so far, no existing animal model can precisely simulate its pathological conditions (Wirtz and Neurath, 2007). Gene-engineered IBD mice models including gene knockout mice, transgenic, and spontaneous colitis models can help assess the efficacy of therapeutic reagent in IBD considering one of its pathogenesis aspects (Lindsay et al., 2003; Jurjus et al., 2004). However, implementation of these models is expensive and time consuming, constraining their application in screening of therapeutic biologics, and inducible colitis models are applied more ubiquitously. Nevertheless, each of these models can only emphasize on one aspect of the mechanism of the disease. It is believed that the DSS-induced colitis model is particularly useful to study the contribution of innate immune mechanisms of colitis (Wirtz and Neurath, 2007) while in chronic TNBS colitis, CD4+ T cells have been shown to play a central role (Neurath et al., 1995). Many of the studies only use one single model to assess the efficacy of the delivery system though distinguished aspects from different models should be taken into consideration. On contrast to some of the current studies, repetitive multiple dosages of biologics are required in clinic for IBD treatment. In our view, acute IBD model accentuate illustration of *in vivo* mechanism of biologics and chronic colitis models are more potent tools to validate biological therapeutic efficacy. Other than *in vivo* mice models, some newly *established in vitro* models demonstrated favorable characteristics in IBD study and can provide patient-specified information in screening of therapeutic biologics. Intestinal organoids are complex three-dimensional structures that mimic the cell-type composition and tissue organization of the intestine derived from the isolation and culture of primary stem cells of intestinal crypts or iPSCs (Serra et al., 2019; Angus et al., 2020). These organoids can be expanded from intestinal tissue samples of healthy and IBD mucosa in both adult and pediatric patient which render improved physiological accuracy model for dissecting IBD pathogenesis and personalized high-throughput screening for new biologics (Dotti and Salas, 2018).

Multiple parameters have settled as standards to assess colitis severity in mice, including clinical activity score assessing weight loss, stool consistency, rectal bleeding, colon length, histological assessment, endoscopy or colonoscopy images, or concentration of multiple cytokines or inflammatory biomarkers such as myeloperoxidase. New evaluation standards can bring innovative perspectives and achieve a better understanding for the biological therapeutic effect. Oral delivered biologics have a potential problem of drug lost in stool yet very rare of researchers have evaluated this aspect. Feces drug concentration can be vital to explain low efficacy of certain oral delivery system for biologics (Brandse et al., 2015). Gut microbiota is a newly recognized factor playing an essential role in IBD development. It was reported that short-term treatment with enterically coated antibiotics dramatically reduced intestinal inflammation. The relevance of microbiota in IBD is also illustrated in a report where reduced microbiota diversity was observed in the fecal microbiome in patients with CD compared with healthy control (Manichanh et al., 2006). The evaluation of the effect of a biological formulation on gut microbiota can provide a unique perspective on their efficacy. Organoid in 2D culture such as the air–liquid interface cultures of the intestinal stem have been used as an established model to study host–microbiota interactions in IBD (Wang et al., 2015; Dotti and Salas, 2018). It will be an illustrative tool for research on formulation-microbiota interaction. Gut-on-chip is another state-in-art invention that can be utilized in IBD therapy (Kim et al., 2016; Poceviciute and Ismagilov, 2019). It can more closely represent the 3D structure and physiological microenvironment of native tissues by incorporating live cells into microfluidic platforms and demonstrate host-pathogen interactions in IBD pathogenesis (Lee et al., 2016; Ashammakhi et al., 2020).

Altogether, colitis targeting *via* oral delivery remains a promising strategy with potential to refine biological therapy in IBD treatment. It may be capable of providing new clinical strategies for biologic delivery due to reduced ADRs and increase local drug concentration. Remaining an unfulfilled task, the development of oral delivery systems for biologics in IBD treatment still requires ingenious inspiration and assiduous study.

## Author Contributions

WZ, CM, and AB designed the structure of the manuscript. WZ and CM wrote the first draft. AB, WZ, and CM corrected and edited the manuscript. All the authors contributed to the article and approved the submitted version.

## Conflict of Interest

The authors declare that the research was conducted in the absence of any commercial or financial relationships that could be construed as a potential conflict of interest.
